# Prolonged Intubation in Patients With Prior Cerebrovascular Disease and COVID-19

**DOI:** 10.3389/fneur.2021.642912

**Published:** 2021-04-09

**Authors:** Shibani S. Mukerji, Sudeshna Das, Haitham Alabsi, Laura N. Brenner, Aayushee Jain, Colin Magdamo, Sarah I. Collens, Elissa Ye, Kiana Keller, Christine L. Boutros, Michael J. Leone, Amy Newhouse, Brody Foy, Matthew D. Li, Min Lang, Melis N. Anahtar, Yu-Ping Shao, Wendong Ge, Haoqi Sun, Virginia A. Triant, Jayashree Kalpathy-Cramer, John Higgins, Jonathan Rosand, Gregory K. Robbins, M. Brandon Westover

**Affiliations:** ^1^Department of Neurology, Massachusetts General Hospital, Boston, MA, United States; ^2^Harvard Medical School, Boston, MA, United States; ^3^Henry and Allison McCance Center for Brain Health, Massachusetts General Hospital, Boston, MA, United States; ^4^Division of Pulmonary and Critical Care Medicine, Massachusetts General Hospital, Boston, MA, United States; ^5^Clinical Data A.I. Center (CDAC), Massachusetts General Hospital, Boston, MA, United States; ^6^Division of General Internal Medicine, Massachusetts General Hospital, Boston, MA, United States; ^7^Department of Psychiatry, Massachusetts General Hospital, Boston, MA, United States; ^8^Department of Pathology, Massachusetts General Hospital, Boston, MA, United States; ^9^Department of Systems Biology, Harvard Medical School, Boston, MA, United States; ^10^Department of Radiology, Massachusetts General Hospital, Boston, MA, United States; ^11^Athinoula A. Martinos Center for Biomedical Imaging, Department of Radiology, Massachusetts General Hospital, Charlestown, MA, United States; ^12^Division of Infectious Diseases, Massachusetts General Hospital, Boston, MA, United States; ^13^The Mongan Institute, Massachusetts General Hospital, Boston, MA, United States

**Keywords:** cerebrovascular disease, COVID-19, respiratory failure, stroke, history of neurological disease, intubation, critical illness, outcomes

## Abstract

**Objectives:** Patients with comorbidities are at increased risk for poor outcomes in COVID-19, yet data on patients with prior neurological disease remains limited. Our objective was to determine the odds of critical illness and duration of mechanical ventilation in patients with prior cerebrovascular disease and COVID-19.

**Methods:** A observational study of 1,128 consecutive adult patients admitted to an academic center in Boston, Massachusetts, and diagnosed with laboratory-confirmed COVID-19. We tested the association between prior cerebrovascular disease and critical illness, defined as mechanical ventilation (MV) or death by day 28, using logistic regression with inverse probability weighting of the propensity score. Among intubated patients, we estimated the cumulative incidence of successful extubation without death over 45 days using competing risk analysis.

**Results:** Of the 1,128 adults with COVID-19, 350 (36%) were critically ill by day 28. The median age of patients was 59 years (SD: 18 years) and 640 (57%) were men. As of June 2nd, 2020, 127 (11%) patients had died. A total of 177 patients (16%) had a prior cerebrovascular disease. Prior cerebrovascular disease was significantly associated with critical illness (OR = 1.54, 95% CI = 1.14–2.07), lower rate of successful extubation (cause-specific HR = 0.57, 95% CI = 0.33–0.98), and increased duration of intubation (restricted mean time difference = 4.02 days, 95% CI = 0.34–10.92) compared to patients without cerebrovascular disease.

**Interpretation:** Prior cerebrovascular disease adversely affects COVID-19 outcomes in hospitalized patients. Further study is required to determine if this subpopulation requires closer monitoring for disease progression during COVID-19.

## Introduction

Disease outcomes associated with the novel coronavirus 2019 (COVID-19) are heterogeneous and include asymptomatic disease, mild respiratory tract illness, severe pneumonia with respiratory failure and acute respiratory distress syndrome, and death (1). It is estimated that one in four patients infected with SARS-CoV-2 requires supplemental oxygen or invasive mechanical ventilation (1–5). To date, survival has been correlated with multiple factors including age, medical comorbidities, and host response to the virus that may lead to multiorgan dysfunction, coagulopathy, and elevated inflammatory markers (1, 4–7).

The prevalence of hospitalized patients with neurological comorbidities COVID-19 widely varies between 1 and 12% depending on the cohort and comorbidities studied (8–15). Our understanding of the risk of COVID-19 critical illness due to chronic neurological conditions remains limited, with cohorts from Asia and Europe suggesting a history of ischemic or hemorrhagic stroke as risk factors for severe events such as mechanical ventilation (MV) (8) and death (8, 10). Recently, two meta-analyses reviewed the relationship between the prior cerebrovascular disease and in-hospital outcomes in COVID-19, using data derived from Chinese and European cohorts, and suggested an increase in risk for critical illness (16) and mortality (17) among patients with prior cerebrovascular disease.

Studies prior to the pandemic suggest that patients with neurological comorbidities are at increased risk for critical illness compared to similarly matched older patients (18, 19). Cerebrovascular comorbidities are common among older adults in the United States (U.S.) where an estimated 3% of adults have had a prior ischemic stroke (7.8 million) (20). A recent study from a U.S. cohort of 3,248 patients suggested an increased odds of in-hospital death among individuals with stroke and COVID-19, however, detailed data on other in-hospital adverse outcomes is largely unknown (21). Given that prior cerebrovascular disease is one of the most common neurological comorbidities in hospitalized patients with COVID-19, information regarding severe outcomes in this population would be valuable for prioritizing prevention strategies in the outpatient neurology setting, providing prognostic information for patients and families, and assisting hospital projections as countries experience increasing numbers of SARS-CoV-2 infections.

In this study, we examined the relationship between prior cerebrovascular diseases and critical illness in the first 28 days of admission and determined the likelihood of successful extubation over a 45-day in-hospital follow-up among adults with positive SARS-CoV-2 RNA admitted to an academic hospital in Boston, Massachusetts, during the first 2 months of the city's outbreak. We hypothesized that prior cerebrovascular disease was a risk factor for critical illness in COVID-19 and a comorbidity associated with increased duration of mechanical ventilation (MV).

## Methods

### Study Setting and Population

**Figure 1:**
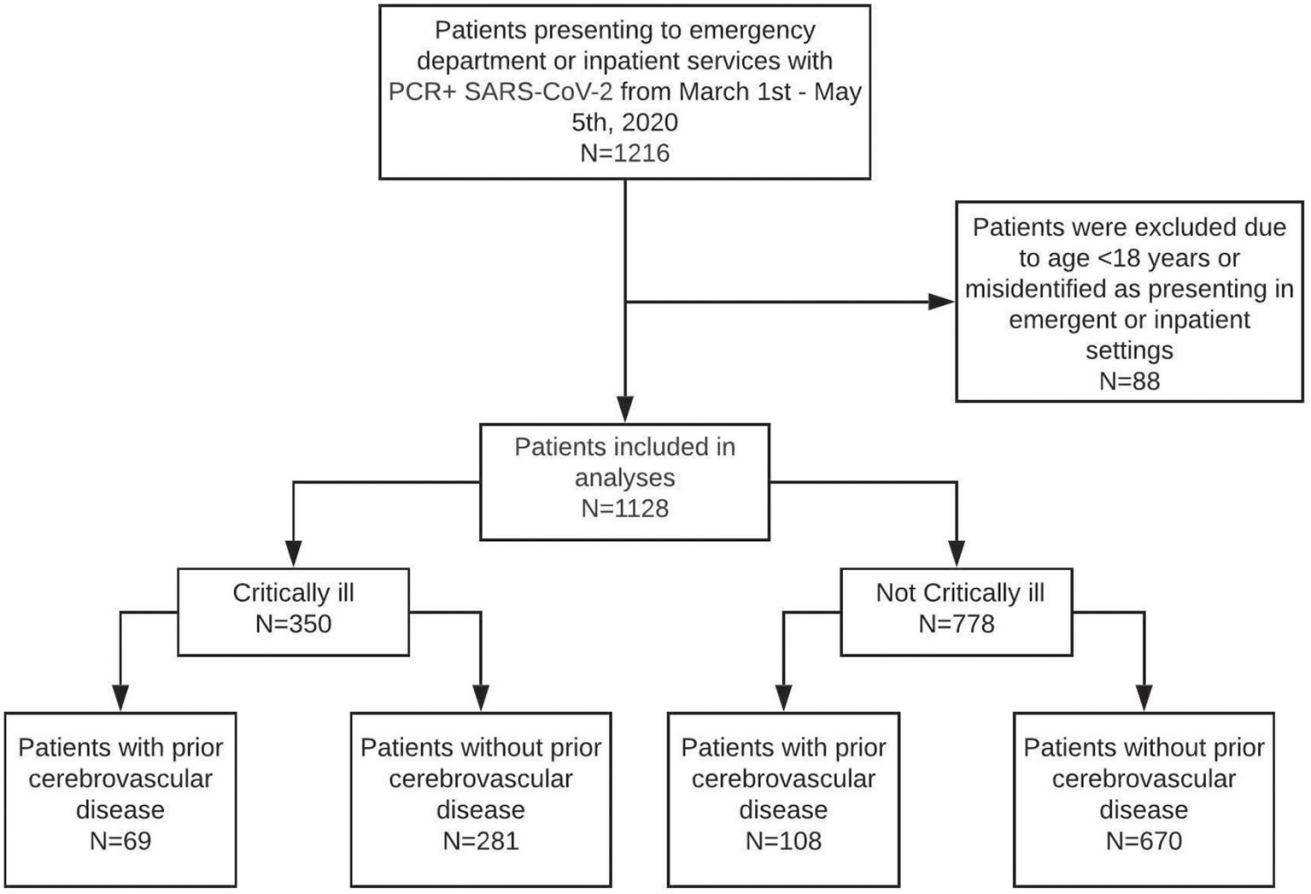
Cohort diagram.

This is an observational study of 1,128 consecutive patients with laboratory-confirmed SARS-CoV-2 infection hospitalized at Massachusetts General Hospital (MGH), a single-center tertiary care facility in Boston, MA. Laboratory confirmation of SARS-CoV-2 infection was obtained using real-time reverse transcription-polymerase chain reaction assays with Food and Drug Administration emergency use authorization. We identified 1,216 patients seen in the Emergency Department (ED) or hospitalized between March 1st and May 5th, 2020, given that the first reported case of SARS-CoV-2 infection in Boston was March 2nd, 2020 (Figure 1). Patients < 18 years old and patients classified as being seen in the ED using electronic data collection but were seen in outpatient clinics after manual review of cases were excluded from analyses. The institutional review board approved this study (Protocol #: 2013P001024) with a waiver of consent for retrospective analyses.

### Data Collection

Engineers from the MGH Clinical Data AI Center extracted COVID-19-related data from the Partners Healthcare Systems Enterprise Data Warehouse, which comprises electronic medical record data from the Mass General Brigham network (formerly Partners Healthcare). Data queried for this study included demographics, admission, discharge, intubation and extubation events, diagnosis and International Classification of Diseases, Tenth Revision, Clinical Modification codes (ICD-10-CM), vital signs, laboratory values within 48 h of admission, and other data. Diagnostic categories were created by grouping ICD-10-CM diagnosis codes using a combination of groupings by SNOMED CT, an ontology-based terminology owned and maintained by the standards development organization SNOMED International and licensed through the National Library of Medicine. Four clinicians performed a manual chart review of identified cases of prior cerebrovascular disease up to 7 days before COVID-19 hospital admission (HA, SM, LB, and AN); a new diagnosis of cerebrovascular disease was recorded if the event occurred within 7 days prior to or after a positive SARS-CoV-2 PCR result.

Of the patients with COVID-19 included in this study, 289 underwent diagnostic neuroimaging after admission (computed tomography or magnetic resonance imaging of the brain), with 484 unique studies performed during the study period. Two radiologists (MDL and MLa) identified evidence of prior intracerebral pathology by reviewing neuroradiographic reports and categorizing intracranial pathology into seven clinical findings: acute or subacute ischemic infarct, chronic ischemic infarct, acute or subacute intracranial hemorrhage, chronic intracranial hemorrhage, post-surgical change (limited to any intracranial surgery), intracranial mass (metastases or primary malignancies), and traumatic brain injury. These measures were used to provide ancillary information on cerebrovascular disease history in addition to ICD-10-CM codes; all positive imaging data not identified using ICD-10-CM codes were manually reviewed to confirm the clinical suspicion of diagnosis (HA and SM), and added 14/289 additional cases.

The date of intubation was obtained using ventilator flowsheets, marking positive end-expiratory pressure (PEEP), and FiO2. Date of extubation was flagged if there were no PEEP and FiO2 readings after 48 h of continuous intubation markings on ventilator flowsheets. Two critical care physicians (HA and LB) manually confirmed intubation in all cases and extubation status and date in 83% of cases (*n* = 243/294). Death and death date was extracted from the EHR. All patients with a date of death were manually reviewed by study team members (AN, LB, SC, and KK) to determine if they transitioned to comfort-measures-only (CMO) and if so, date of transition was recorded.

### Exposure and Outcome Measures

The binary variable for cerebrovascular history included at least one diagnosis of ischemic stroke, intracerebral hemorrhage (ICH), venous sinus thrombosis (VST), subarachnoid hemorrhage (SAH), or subdural hemorrhage (SDH) recorded at least 7 days prior to admission. A composite outcome, critical illness (not to be confused with critical illness myopathy), was defined as invasive MV or death within 28 days of admission (4), and used for the primary analysis. A secondary outcome was the probability of successful extubation, defined as liberation from mechanical ventilation irrespective of the mode of ventilation delivery (i.e., endotracheal or tracheostomy tube). Follow-up time was right-censored on June 20th, 2020, to allow 45 days of observation for intubated patients given long-durations of intubation in COVID-19 patients. All positive prior neurological diagnoses and outcomes were confirmed manually by chart review (HB, SM, AN, LB, SC, and KK).

### Statistical Analysis

Continuous and categorical variables were presented as median [interquartile range, (IQR)] and n (%), respectively. Mann-Whitney U tests, χ^2^ tests, or Fisher's exact tests were used to compare differences between critical and non-critical patients where appropriate.

The control for confounding was done using inverse probability weighting (IPW) method. The propensity scores for cerebrovascular disease were estimated with a multivariable logistic regression model that included age, sex, Latinx ethnicity, and Black or African American race. The predicted probabilities from this propensity-score model were used to calculate the stabilized IPW scores (22). Logistic regression models using IPW are reported, and models estimated odds ratios (OR) [95% confidence intervals (CI)] for the association between history of cerebrovascular disease and critical illness by day 28. In sensitivity analyses, a matching strategy was used to match patients with and without cerebrovascular disease in a 1:2 ratio by age, sex, Latinx, and Black or African American race. The smallest average absolute distance was used to match across all pairs (R 4.0.0 MatchIt package).

We examined the effect of cerebrovascular disease history on the duration of intubation, with death as a competing event. Time was measured in days from intubation to successful extubation or death (two mutually exclusive events) and censored at 45 days if no event was observed. If a patient was extubated and died during the observation period, the event recorded was death. We estimated the cause-specific hazard of transitioning from intubation to successful extubation and calculated the hazard ratio (HR) for patients with cerebrovascular history compared to those without using a propensity score weighted Cox proportional hazards model. The cumulative incidence curves (CICs) for extubation and death were computed using the R package *causalCmprsk* with both Cox PH and Aalen-Johansen's non-parametric estimators (23, 24). Propensity score weights were used for generating the CIC curves to account for confounding by age and sex. We estimated exposure effect as the restricted mean time difference, which is the area under the CIC curve and provides a more clinically meaningful measure than HR (25).

## Results

### Clinical Characteristics of Patients Admitted With COVID-19

A total of 1,128 adult patients were seen in the emergency department or admitted between March 10th and May 5th, 2020, with laboratory-confirmed SARS-CoV-2 RNA infection (Figure 1). The median age of patients was 59 years old (IQR 45–73; range 18–103), 640 (57%) were men, and 401 (36%) were Latinx ethnicity. Chronic medical illnesses were common with 362 (32%) diagnosed with hypertension, 208 (18%) with diabetes, 113 (10%) with renal disease, and 115 (10%) with heart failure (Table 1).

**Table 1:** Baseline characteristics of patients hospitalized with COVID-19.

	**Overall (*n =* 1,128)**	**Critically Ill (*n =* 350)**	**Not Critically Ill (*n =* 778)**	***P*-value, Critically ill vs. Not Critically Ill**
**DEMOGRAPHICS**, ***N (%)***				
Age, median [IQR]	59 [45, 73]	65.00 [53, 77]	56.00 [42, 70]	<0.001^*^
18–29	59 (5.2)	7 (2.0)	52 (6.7)	
30–39	125 (11.1)	22 (6.3)	103 (13.2)	
40–49	168 (14.9)	42 (12.0)	126 (16.2)	
50–59	227 (20.1)	63 (8/0)	164 (21.1)	
60–69	207 (18.4)	76 (21.7)	131 (16.8)	
70–79	177 (15.7)	72 (20.6)	105 (13.5)	
80–89	129 (11.4)	55 (15.7)	74 (9.5)	
≥ 90	36 (3.2)	13 (3.7)	23 (3.0)	
Sex				0.003^*^
Female	488 (43.3)	128 (36.6)	360 (46.3)	
Male	640 (56.7)	222 (63.4)	418 (53.7)	
Race				
African American or Black	137 (12.1)	44 (12.6)	93 (12.0)	0.845
Asian	41 (3.6)	13 (3.7)	28 (3.6)	1.000
White	467 (41.4)	147 (42.0)	320 (41.1)	0.835
Latinx Ethnicity	401 (35.5)	110 (31.4)	291 (37.4)	0.008^*^
Tobacco Use				<0.001^*^
Never	645 (57.2)	168 (48.0)	477 (61.3)	
Former	218 (19.3)	81 (23.1)	137 (17.6)	
Present	96 (8.5)	25 (7.1)	71 (9.1)	
Not asked	45 (4.0)	10 (2.9)	35 (4.5)	
**MEDICAL COMORBIDITIES**, ***N (%)***				
Charlson Comorbidity Index Score, mean (SD)	1.53 (2.24)	1.83 (2.47)	1.39 (2.12)	0.002^*^
0 comorbidities	562 (49.8)	157 (44.9)	405 (52.1)	
1–2 comorbidities	307 (27.2)	93 (26.6)	214 (27.5)	
≥ 3 comorbidities	259 (23.0)	100 (28.6)	159 (20.4)	0.008^*^
Hypertension	362 (32.1)	121 (34.6)	241 (31.0)	0.260
Diabetes	204 (18.1)	72 (20.6)	132 (17.0)	0.170
Myocardial infarction	33 (2.9)	14 (4.0)	19 (2.4)	0.213
Congestive heart failure	115 (10.2)	46 (13.1)	69 (8.9)	0.037^*^
Chronic obstructive pulmonary disease	154 (13.7)	49 (14.0)	103 (13.5)	0.893
Renal disease	113 (10.0)	52 (14.9)	61 (7.8)	<0.001^*^
Peripheral vascular disease	87 (7.7)	41 (11.7)	46 (5.9)	0.001^*^
Body Mass Index, median [IQR]	28.8 [25.3, 33.3]	28.9 [25.3, 34.1]	28.8 [25.2, 32.9]	0.450
**NEUROLOGIC COMORBIDITIES**, ***N (%)***				
Cerebrovascular comorbidities	177 (15.7)	69 (19.7)	108 (13.9)	0.016^*^
Acute Ischemic Stroke	112 (9.9)	49 (14.0)	63 (8.1)	0.016^*^
Venous Sinus Thrombosis	4 (0.4)	3 (0.9)	1 (0.1)	0.173
Subarachnoid Hemorrhage	5 (0.4)	3 (0.9)	2 (0.3)	0.358
Subdural Hemorrhage	60 (5.3)	18 (5.1)	42 (5.4)	0.973
Intracerebral Hemorrhage	22 (2.0)	11 (3.1)	11 (1.4)	0.087
Other neurological comorbidities				
Dementia	39 (3.5)	15 (4.3)	24 (3.1)	0.398
Movement disorder	28 (2.5)	10 (2.9)	18 (2.3)	0.737
Neuromuscular disorder	56 (5.0)	22 (6.3)	34 (4.4)	0.222
Seizure history	25 (2.2)	6 (1.7)	19 (2.4)	0.583
Brain tumor	8 (0.7)	1 (0.3)	7 (0.9)	0.451

* *indicates a p < 0.05. Charlson Comorbidity Index Score includes acute myocardial infarction, congestive heart failure, peripheral vascular disease, cerebrovascular disease, dementia, chronic obstructive pulmonary disease, rheumatoid disease, peptic ulcer disease, liver disease, diabetes with and without complications, hemiplegia or paraplegia, renal disease, cancer, metastatic solid tumor, and HIV/AIDS*.

**Figure 2:**
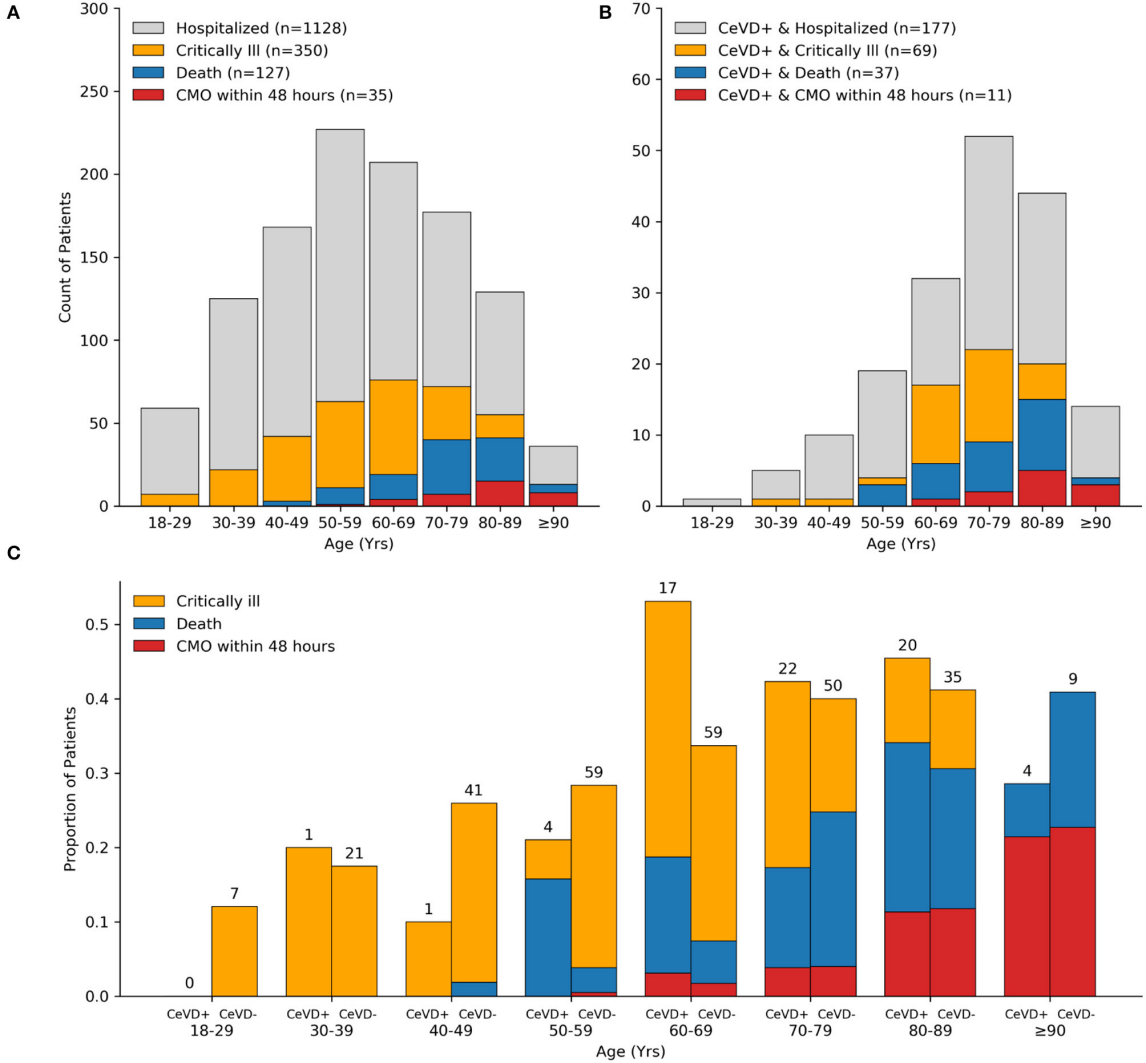
Distribution of COVID-19 severe outcomes by cerebrovascular disease history. Bar plots showing counts of all hospitalized patients **(A)** and cerebrovascular disease subset **(B)** with the indicated COVID-19 outcome stratified by decade of life. The majority of critically ill patients over age 70 years and 100% of patients age 90 years old or older died within 28 days of admission. Age distribution of patients with cerebrovascular disease and COVID-19 was left-skewed compared to the total cohort. Bar plots showing the proportion of critically ill patients who died stratified by cerebrovascular disease status and decade of life. Counts (top of bars) show the number of critically ill patients in respective age groups and cerebrovascular disease status **(C)**. CeVD, cerebrovascular disease; CMO, comfort measures only.

By day 28 of admission, 350 patients (31%) became critically ill, including 127 (11%) who died (Table 1). Most patients were intubated within 24 h of admission (median 0 days, IQR 0–3 days). The median time to death among those hospitalized was 9.5 days (IQR 4–17), and most deaths occurred in patients over 70 years old, and 28% (35/127) were transitioned to comfort measures only (CMO) within 48 h of admission (Figure 2A). Mortality among those intubated was 24% (70/293). Across ethnic and racial groups, critical illness occurred in 44 of 137 (32%) Black or African American patients, 147 of 467 (31%) white patients, and 110 of 401 (27%) patients of Latinx ethnicity.

### Odds of Critical Illness in Patients With Prior Cerebrovascular Disease

A history of ischemic stroke (*n* = 112; 9.9%), ICH (*n* = 22; 2.0%), or SDH (*n* = 60; 5.3%) was frequent among COVID-19 patients with a total of 177 (16%) patients having at least one prior cerebrovascular disease diagnosis. Patients with a prior cerebrovascular disease were more likely to be critically ill compared to non-critically ill individuals [69/350 (20%) vs. 108/778 (14%), *p* = 0.02].

Compared to patients without a cerebrovascular history, individuals with a prior cerebrovascular were older [median 74 [63, 82] vs. 56 [43, 69], *p* < 0.001], more likely to have a history of current use tobacco [71/177 (40%) vs. 243/951 (26%), *p* < 0.001], and more likely to have a higher burden of medical comorbidities as measured by Charlson Comorbidity Index ≥ 3 [87/177 (49%) vs. 172/951 (18%), *p* < 0.001]. A high proportion of patients with cerebrovascular disease ages 50–69 years old died (Figures 2B,C), and across all ages, in-hospital 28-day mortality was higher between patients with vs. without cerebrovascular disease [37/177 (20.9%) vs. 90/951 (9.5%), *p* < 0.001]. The proportion of patients mechanically ventilated or who died, stratified by history of cerebrovascular disease subtype, are detailed in Supplementary Table 1. A total of 39/1,128 (3.5%) patients had a new cerebrovascular event after their COVID-19 diagnosis (7 patients had two or more events), of which 22/39 (56%) had a past history of cerebrovascular disease.

Given prior reports of abnormal inflammatory and thromboembolic indices in patients with a history of ischemic stroke (8), common laboratory markers tested in COVID-19 were assessed on admission. Patients with cerebrovascular disease had higher admission median levels of D-dimer, troponin (*p* < 0.001 for both) and prothrombin time (*p* < 0.01), but minimally lower levels of inflammatory markers such as CRP [63.90 [23.50, 133.30] vs. 75.15 [36.2, 146.1], *p* < 0.01] and ferritin [442 [193.0, 872.0] vs. 547 [286.3, 1051.3], *p* < 0.01] compared to patients without cerebrovascular disease (Table 2). While there were no statistical differences in absolute lymphocyte counts (*p* = 0.3) or platelet levels (*p* = 0.06), patients with cerebrovascular disease had slightly higher red cell distribution widths (RDW) [14.10 [13.00, 15.33] vs. 13.20 [12.60, 14.10], *p* < 0.001], a measure associated with all-cause mortality (26, 27).

**Table 2:** Lab values of patients with COVID-19 stratified by cerebrovascular disease.

**Lab values [median [IQR]]**	**Cerebrovascular History (*n* = 177)**	**No Cerebrovascular History (*n* = 951)**	***P-*value**
**CBC**			
WBC	6.64 [4.95, 9.04]	6.50 [5.05, 8.65]	0.856
ALYMPH	0.96 [0.67, 1.41]	1.00 [0.69, 1.39]	0.310
ANEUT	4.67 [2.98, 6.65]	4.87 [3.45, 6.79]	0.458
HGB	12.45 [10.90, 13.70]	13.40 [12.20, 14.60]	<0.001^*^
RDW	14.10 [13.00, 15.33]	13.20 [12.60, 14.10]	<0.001^*^
PLT	189.50 [148.75, 252.25]	204.00 [158.00, 259.75]	0.063
**INFLAMMATORY**			
D-DIMER	1292.00 [773.00, 2243.50]	968.00 [619.50, 1635.00]	<0.001^*^
FERRITIN	442.00 [193.00, 872.00]	547.00 [286.25, 1051.25]	0.008^*^
IL-6	18.40 [7.80, 55.50]	21.00 [10.50, 40.15]	0.633
CRP	63.90 [23.50, 133.30]	75.15 [36.20, 146.10]	0.005^*^
ESR	39.00 [22.00, 67.00]	39.00 [23.00, 60.00]	0.484
LDH	284.00 [226.00, 373.00]	318.00 [245.00, 424.00]	0.002^*^
**COAGULATION FACTORS**			
PT	14.30 [13.50, 15.50]	13.70 [13.20, 14.53]	<0.001^*^
PT.INR	1.10 [1.00, 1.20]	1.10 [1.00, 1.20]	<0.001^*^
PTT	35.90 [32.25, 40.70]	34.10 [31.00, 38.80]	0.008^*^
**CHEMISTRY**			
AST	37.00 [23.75, 51.25]	39.00 [27.00, 59.00]	<0.001^*^
ALT	22.00 [15.00, 36.00]	30.00 [19.00, 51.00]	<0.001^*^
BILI, TOTAL	0.40 [0.30, 0.62]	0.45 [0.30, 0.60]	0.040^*^
CREATININE	1.06 [0.83, 1.71]	0.89 [0.74, 1.10]	<0.001^*^
eGFR	59.00 [32.00, 81.00]	85.00 [61.25, 101.00]	<0.001^*^
LDH	284.00 [226.00, 373.00]	318.00 [245.00, 424.00]	0.002^*^
**METABOLIC**			
TOTAL CHOL	130.00 [106.00, 159.00]	131.00 [109.00, 151.00]	0.682
HBA1C	6.50 [6.00, 8.00]	6.50 [5.90, 8.20]	0.731

* *indicates a p < 0.05. WBC, White Blood Cell; Alymph, Lymphocyte count; ANEUT, Neutrophil count; HGB, Hemoglobin; RDW, Red blood cell distribution; PLT, Platelet; IL-6, Interleukin-6; C.P.R., C-reactive protein; ESR, Erythrocyte sedimentation rate; LSH, Lactic acid dehydrogenase; PT, Prothrombin Time; INR, International normalized ratio; PTT, Partial thromboplastin time; AST, Aspartate transaminase; ALT, Alanine Transaminase; BILI, Bilirubin; eGFR, eGFR glomerular filtration rate; HBA1C, Hemoglobin A1c*.

In unadjusted analyses, the odds of critical illness were higher in patients with cardiovascular disease compared to patients without cerebrovascular disease [OR 1.5; 95% CI [1.09–2.12]]. In multivariable analyses with IPW according to the propensity score, cerebrovascular disease remained independently associated with critical illness [adjusted OR 1.54, 95% CI [1.14–2.07]] (Table 3). A subsequent sensitivity analysis that used a 2:1 matching ratio (no cerebrovascular disease: cerebrovascular disease) yielded similar results [OR 1.58, 95% CI [1.08–2.36]].

**Table 3:** Associations between cerebrovascular disease history and critical illness.

**Number of events/number of patients at risk (%)**	
Prior cerebrovascular disease	69/177 (39.9%)
No prior cerebrovascular disease	281/951 (29.5%)
	**Odds of critical illness: OR [95% CI]**, ***P-*****value**
Unadjusted	1.52 [1.09–2.12], 0.01
IPW adjusted^α^	1.54 [1.14–2.07], < 0.01
2:1 PS matched^β^	1.58 [1.08–2.36], 0.02

α *the odds ratio was calculated for age, sex, Latinx ethnicity, and Black or White race using an inverse propensity-score weighted IPW logistic regression analysis. The analysis included all 1,128 patients*.

β *sensitivity analyses calculating the odds ratio from 2:1 propensity score (PS) matched cohort. Analyses include 531 cases without cerebrovascular disease history and 177 cases with cerebrovascular disease*.

### Cumulative Incidence of Successful Extubation in Patients With Cerebrovascular Disease

**Figure 3:**
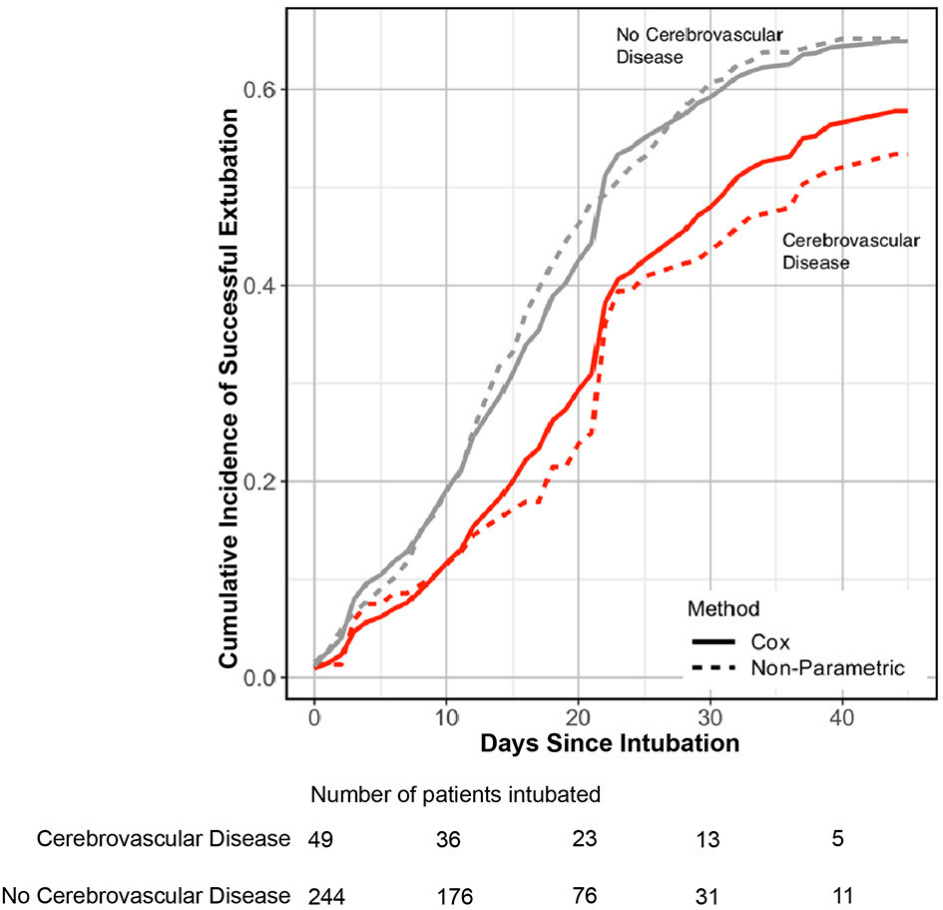
Incidence of successful extubation over 45-days of in-hospital observation. Cox proportional (line) and non-parametric (dashed line) estimation of cumulative incidence of transitioning from intubation to extubation without death in patients with cerebrovascular disease (red) and no cerebrovascular disease history (gray). Patients with cerebrovascular disease had lower cumulative incidence of successful extubation without death over a 45-day observation window [adjusted cause-specific HR 0.57 [95% CI 0.33–0.98]]. The number of patients intubated is shown on the bottom stratified by presence or absence of cerebrovascular disease. CI, Confidence interval; HR, Hazard ratio.

To further understand the relationship between prior cerebrovascular disease and COVID19 severe outcomes, we used a competing risk analysis framework to determine the relationship between cerebrovascular disease and duration of intubation without subsequent death. The cumulative incidence of successful extubation without death in patients with cerebrovascular disease was lower compared to those without cerebrovascular history (Figure 3), and there was a significant association between prior cerebrovascular disease and likelihood of successful extubation (adjusted cause-specific HR, 0.57; 95% CI, 0.33–0.98). Over a 45-day observation window, patients with cerebrovascular disease had a longer intubation time with a restricted mean time difference of 4.02 days [0.34, 9.32] added time on mechanical ventilation compared to patients without cerebrovascular disease using a Cox-PH model. The time difference was modeled using non-parametric cumulative incidence functions in sensitivity analyses and showed an additional 5.65 days [2.40, 10.85] of intubation time in patients with cerebrovascular disease. There were no differences in cause-specific HR for death between patients with and without cerebrovascular history after adjustments.

## Discussion

In this observational study, we present one of the largest analyses with extended follow-up among US hospitalized patients with prior cerebrovascular disease and COVID-19. Of the 1,128 hospitalized patients, 16% (177/1,128) had at least one diagnosis of cerebrovascular disease prior to COVID-19, and odds of critical illness in this subpopulation were 1.5-times higher compared to those without cerebrovascular disease. Additionally, these data show that patients with cerebrovascular disease were less likely to achieve successful extubation and estimated to be ventilated for 4–5 days longer than patients without a prior cerebrovascular disease. Given that available data on cerebrovascular disease comorbidity and in-hospital outcomes are limited, we anticipate these findings to be relevant for outpatient prevention strategies and prognostic discussions with patients and families, especially as countries experience resurges of SARS-CoV-2 infections.

In this cohort, the majority of patients with a prior cerebrovascular disease had a prior acute ischemic stroke (9.9%; 11/1,128), a prevalence which was higher than expected from U.S. 2013–2016 stroke estimates of 2.5% for adults ages > 20 years old (14). These results are consistent with published cohorts suggesting a greater number of hospitalized COVID-19 patients with cerebrovascular disease than prevalence estimates and increased likelihood of negative outcomes (4, 8, 10–13, 16, 17, 21, 28–31). The findings presented here extends our understanding of comorbidities that may contribute to increased risk of critical illness in COVID-19, and additionally suggests that critically ill COVID-19 patients with cerebrovascular disease may be prone to longer mechanical ventilation time than patients of similar ages and demographics without cerebrovascular disease. It is possible that patients with prior history of cerebrovascular disease are vulnerable to prolonged ventilation in COVID-19 given a propensity for lower levels of premorbid function, increased probability of cerebrovascular events after diagnosis or increased risk of frailty, a clinical state which is a strong predictor of adverse health effects including hospitalization, disability, and mortality (32). Recent data also suggests that COVID-19 patients with delirium are more likely to have longer duration of intubation (33). Given that predictors of delirium include medical comorbidities such as cerebrovascular disease, it may be challenging to distinguish which factors predominate in conferring risk of longer intubation times and requires large scale studies. Irrespective of cause, the impact of longer duration of intubation can be extrapolated from other critical care studies prior to the COVID-19 pandemic, which show that increased ventilation times are associated with a greater need for sedation and analgesics and higher rates of ventilator-associated pneumonia, line infections, urinary tract infections, delirium, ileus, and decubitus ulcers (34–36). Further, additional days on ventilators are likely to lead to greater rehabilitation needs, reduced cognitive function, and could be uniquely detrimental for patients with prior neurological deficits and their family members (37, 38).

A prior study suggested that patients with cerebrovascular disease may have more aggressive inflammatory responses on admission for COVID-19 (8). While our data did not show increases in CRP or ferritin or evidence of lymphopenia among patients with a prior cerebrovascular disease, they had higher levels of D-dimer and troponin levels, consistent with a prior study of stroke patients (8). Additionally, admission RDW was elevated in patients with cerebrovascular disease in this study. Elevated RDW has been shown to be a marker of all-cause mortality, a predictor of complicated hospitalizations that included the need for MV from infectious causes such as influenza (26, 39). and recent data suggests an association with increased mortality risk in COVID-19 (40). Given that COVID-19 is associated with diffuse coagulopathy and thrombotic events (41–43). further study is required to determine if COVID-19 infection exacerbates vascular pathology present in patients with cerebrovascular disease and if this subpopulation requires closer diagnostic monitoring for coagulopathy and disease progression during COVID-19.

Our work has several limitations worth noting. It is a single-center observational study and relies on the EHR, which may not capture full medical histories; thus, some misclassification of prior medical diagnosis is possible. To minimize misclassification bias, our group manually validated data relying on expertise from clinicians in multiple disciplines. We allowed for ancillary data regarding history of cerebrovascular disease based on radiographic imaging data to be introduced, and while we added only a small fraction of patients to the overall cohort with history of cerebrovascular disease (14 patients), this may have led to residual confounding. Data on premorbid level of functioning and details of prior lung function were unavailable and could impact the likelihood of successful extubation; neither could be adjusted for in our analyses. Long-term follow-up that includes cognitive assessments for patients with prior cerebrovascular disease will be critical to understand the longitudinal impact of COVID-19 in this subgroup. Finally, we used SARS-CoV-2 RT-PCR positive results as an indicator of COVID-19 disease, however, PCR results may be an incidental finding in some cases. At the time of admission for this cohort, asymptomatic patients were not routinely tested using SARS-COV-2 RT-PCR, and the majority of cases presented were clinically considered as having COVID-19.

In summary, our findings show that patients with cerebrovascular disease and COVID-19 have higher odds of critical illness, and a lower incidence of successful extubations. This subpopulation is estimated to have longer mechanical ventilation times compared to patients of similar ages without cerebrovascular disease. In aggregate, these data suggest there are important opportunities for proactive outpatient neurological care and open discussion regarding vaccine allocation priorities and for the management and expectations of duration of mechanical ventilation and critical disease in patients with cerebrovascular disease.

## Data Availability Statement

The raw data supporting the conclusions of this article will be made available by the authors, without undue reservation.

## Ethics Statement

The studies involving human participants were reviewed and approved by Mass General Brigham (MGB) institutional review board (IRB). Written informed consent for participation was not required for this study in accordance with the IRB for retrospective analyses.

## Author Contributions

SM, SD, HA, GR, and MW: had full access to all the data in the study and take responsibility for the integrity of the data and the accuracy of the data analysis. SM, SD, HA, JR, GR, and MW: concept and design. SM, SD, HA, LB, SC, EY, KK, and CB: drafting of the manuscript. SM, SD, HA, LB, BF, JH, MDL, ML, VT, JR, GR, and MW: critical revision of the manuscript for important intellectual content. SM, SD, CM, HS, MJL, AJ, WG, Y-PS, EY, and MW: statistical analysis. SM, SD, HA, GR, and MW: supervision. All authors: acquisition, analysis, or interpretation of data.

## Conflict of Interest

JR is supported by OneMind and the American Heart Association and has consulted for Boehringer Ingelheim, outside the scope of this work. MA is co-founder, equity holder, and has consulted for Day 0 Diagnostics, outside the scope of this work. LB has research funding from Apple Inc., outside the scope of this work. JK-C has research funding from General Electric, outside the scope of this work. GR reports grants paid to MGH from Pfizer, Gilead, Citius Pharm, Emergent Biosolutions, Leonard Meron Bioscience, for clinical trial support outside the submitted work. The remaining authors declare that the research was conducted in the absence of any commercial or financial relationships that could be construed as a potential conflict of interest.
