# Supplementary material for: Prolonged Intubation in Patients With Prior Cerebrovascular Disease and COVID-19
Source: Front Neurol. 2021 Apr 9;12:642912. doi: 10.3389/fneur.2021.642912 (PMC8062773; doi:10.3389/fneur.2021.642912)
Supplement: Supplementary file 1 [file Table_1.docx]

**Supplementary Table 1: Proportion of patients mechanically ventilated and / or dead by hospital day 28 stratified by acute cerebrovascular disease (CVD) and history of CVD subtypes**

| Day 28 Outcome |  | History of CVD Subtype | | | | | |
| --- | --- | --- | --- | --- | --- | --- | --- |
| All, n (%) | Acute Post-COVID-19 CVD  (n = 39) | Acute Ischemic Stroke  (n = 112) | Intracerebral Hemorrhage  (n = 22) | Subarachnoid Hemorrhage  (n = 5) | Subdural Hemorrhage  (n = 60) | Venous Sinus Thrombosis  (n = 4) | Multiple CVD Diagnosis  (n = 22) |
| Mechanical Ventilation, n (%) | 23 (59.0) | 33 (29.5) | 9 (40.9) | 1 (20) | 17 (28.3) | 2 (50) | 13 (30.8) |
| Dead, n (%) | 9 (23.1) | 30 (26.8) | 4 (18.2) | 3 (60) | 8 (13.3) | 2 (50) | 7 (54) |
| Duration of Intubation, days  mean (standard deviation) | 16.43 (9.83) | 15.32 (12.50) | 16.78 (15.75) | 0 (0)^a^ | 15.88 (17.41) | 8.5 (11.31) | 9.42 (11.94) |

^a^ Duration of intubation < 24 hours
